# Frequency of Iatrogenic Sexual Dysfunction Associated with Antihypertensive Compounds

**DOI:** 10.3390/jcm10225214

**Published:** 2021-11-09

**Authors:** Bárbara Buch-Vicente, José Mª. Acosta, José-Angel Martín-Oterino, Nieves Prieto, María Elena Sánchez-Sánchez, Purificación Galindo-Villardón, Angel L. Montejo

**Affiliations:** 1 Instituto Investigación Biomédica de Salamanca (IBSAL), Paseo San Vicente SN, 37007 Salamanca, Spain; barbarabuch@usal.es (B.B.-V.); nievesprieto2020@gmail.com (N.P.); elenamerysr@gmail.com (M.E.S.-S.); amontejo@usal.es (A.L.M.); 2 Internal Medicine Service, Clinical Hospital of Salamanca, Paseo San Vicente SN, 37007 Salamanca, Spain; jmoterino@saludcastillayleon.es; 3 Psychiatry Service, Clinical Hospital of Salamanca, Paseo San Vicente SN, 37007 Salamanca, Spain; 4 Statistical Department, Campus Miguel de Unamuno, University of Salamanca, Calle Alfonso X El Sabio s/n, 37007 Salamanca, Spain; pgalindo@usal.es; 5 Centro de Investigación Institucional, Universidad Bernanrdo O’Higgins, Av. Viel 1497, Santiago 8320000, Chile; 6 Department of Psychiatry, Nursing School, University of Salamanca, Av. Donantes de Sangre SN, 37007 Salamanca, Spain

**Keywords:** sexual dysfunction, antihypertensive treatment, hypertension, PRSexDQ-SALSEX, blood pressure

## Abstract

Iatrogenic sexual dysfunction (SD) caused by antihypertensive (AH) compounds, provoking sexual desire, orgasm or arousal dysfunction, is a common clinical adverse event. Unfortunately, it is often underestimated and underreported by clinicians and prescribers in clinical practice, deteriorating the adherence and patient quality of life. The objective of this study was to investigate the frequency of SD in patients treated with different antihypertensive compounds; a real-life naturalistic and cross-sectional study in patients receiving AH treatment was carried out. Method: A total of 256 patients were included in the study (188 males and 68 females who met the inclusion and exclusion criteria). The validated Psychotropic-Related Sexual Dysfunction Questionnaire (PRSexDQ-SALSEX) was transversally applied once at least every two months following the onset of the treatment in order to measure possible AH-related SD. Although the spontaneous reporting of SD was very low (6.81% females/24.8% males), 66.40% of the patients reported impaired sexual function through the SALSEX questionnaire after the treatment onset, as follows: decreased desire (55.8% females/54.2% males), delayed orgasm (42.6%/45.7%), anorgasmia (42.6%/43.6%) and arousal difficulties (53%/59.6%). The average frequency of moderate to severe iatrogenic SD was 66.4% with AH in monotherapy as follows: angiotensin II receptor antagonists (ARBs), 29.8%; calcium antagonists, 40%; diuretics, 42.9%; beta blockers, 43.8%; and angiotensin-converting enzyme (ACE) inhibitors, 77.8%. Combined treatments showed a higher percentage of main SD (70.3%): diuretic + ACE inhibitor, 42.3%; ARB + calcium antagonist, 55.6%; diuretic + calcium antagonist, 68.8%; and diuretic + ARB, 74.2%. The greatest risk factors associated with SD were poor general health, age over 60 with a comorbid coronary or musculoskeletal disease, mood disorder and diuretic +ARB combined therapy. Conclusion: SD is common in patients treated with antihypertensive drugs, and it is still underreported. The most harmful treatment deteriorating sexual function was the combination of diuretic +ARB, while the least harmful was monotherapy with ARBs. More research is needed on the clinical management of this problem to preserve the quality of life of patients and their partners.

## 1. Introduction

Hypertension (HT) is a disease recognized for its increasing incidence and difficult management. This long-term disease causes severe associated pathologies, and these are the main cause of premature death worldwide [1]. Since hypertension is a disease that can be prevented and controlled, the availability of antihypertensive drugs in addition to the acquisition of healthy lifestyle habits should be first-order health objectives, as indicated by the WHO in its HEARTS program launched to manage hypertension from primary health care [2]. Antihypertensive drugs are frequently associated with undesirable side effects that reduce patient quality of life, compromising adherence to treatment. Hypertension, similar to other chronic diseases, has been reported to be associated with poor adherence to treatment [3].

Sexual dysfunction (SD) is one of the most frequent adverse effects in patients with HT who take antihypertensive drugs [4,5], especially males, and often persists throughout the disease [6,7]. The most common symptom is erectile dysfunction, which has been widely studied in men [8,9]. However, other aspects involved in sexual function such as desire, orgasm and arousal difficulties in women have been poorly recognized to date. Although SD can be caused by the disease itself and the pathophysiological mechanisms involved (e.g., vascular stiffness and arteriosclerosis), several studies have shown that antihypertensive treatments can cause the worsening of sexual function (10), especially diuretics and non-cardio-selective beta blockers [4,5,10,11]. However, studies with recent drugs such as angiotensin II receptor blockers show less impairment of sexual function, including erectile dysfunction. The results described in the literature on the frequency of sexual problems associated with antihypertensives are inconsistent and sometimes contradictory and should still be interpreted with caution [12] as there is a great deal of variability in associated variables such as concomitant diseases, age differences, gender and previous sexual function status [6]. On the other hand, the results on associated sexual dysfunction have focused on monotherapy without including the most recent drugs or combination therapies, making it difficult to draw conclusions for routine clinical practice. Moreover, an additional problem in previous methodological designs could be that other physical illnesses and concomitant treatments that may influence sexual response are not considered. In addition, patients generally do not spontaneously report these difficulties to their doctors, and in turn, their doctors usually do not make a specific exploration of the issue due to embarrassment or inexperience in the field of sexuality. The absence of inclusion and exclusion criteria in most studies limits the generalizability of results, and new data on the real frequency associated with the use of antihypertensive drugs and their pharmacological associations should be obtained, removing confounding factors as much as possible. On the other hand, there are no specific scales to measure sexual dysfunction associated with antihypertensive medication use.

The primary objective of this study was to describe the prevalence of sexual dysfunction in patients diagnosed with hypertension treated with single or combined antihypertensive therapy. Other objectives were to relate the effect of SD to adherence to treatment and to identify other clinical and pharmacological variables that might impair sexual function in hypertensive subjects on antihypertensive treatment.

## 2. Experimental Section

### 2.1. Design

A multi-centric, transversal, epidemiological and observational study was carried out. This study measured the prevalence of SD in patients treated with antihypertensives to explain the variability in SD that has been observed in patients being treated with the different compounds, employed either alone or in combination.

### 2.2. Study Population

This study included 256 adult out-patients (188 males and 68 females) aged from 33 to 98 years (median = 58 years; 24.8% < 50 years old; 31.9%—51–60 y.o.; 28.7%—61–70 y.o.; and 14.6% > 70 y.o., with only 6 subjects > 80 y.o.) with a high blood pressure (HBP) diagnosis, in treatment with either one antihypertensive agent alone or with multiple in combination, treated for at least two months prior to inclusion in the study, in a primary care facility or specialized unit. Inclusion criteria: normal sexual function prior to antihypertensive treatment, sexually active at the time of inclusion into the study, with previously habitual and satisfactory sexual practices. All patients signed an informed consent form. Exclusion criteria: subjects suffering from abuse or dependency on alcohol and/or narcotics, serious and poorly controlled medical illnesses known to interfere with sexual function (cardiopathy, diabetes, kidney or liver failure, degenerative illnesses, etc.); ongoing hormone treatment (2 months prior to inclusion in the study) or any other medication that would interfere with sexual intercourse (hypoglycemics, antiepileptics, H2-antagonists, opiates, antidepressants, antipsychotics); mental deficiency, or the inability to understand the study’s procedures. Most subjects were married with a stable partner with whom they were able to have sexual intercourse (80.4%). The remaining sample (19.6%) were distributed among single (6.9%), single with a stable partner (3.2%), widowed (3.7%) and divorced (5.8%).

### 2.3. Method

Data collection was conducted through two empirical procedures, as follows.

Clinical interview: This was conducted by the patient’s usual physician or nurse. The following information was obtained during the clinical interview: a checklist of inclusion and exclusion criteria; the signing of informed consent forms; clinical and socio-demographic data; pharmacologic treatment for hypertension, concomitant medication and any relevant illnesses; questions about previous sexual conduct; and perception of their general state of health and sexual function both before and after beginning the antihypertensive treatment.

Main variable: Sexual function was evaluated with the validated Psychotropic-Related Sexual Dysfunction Questionnaire (PRSexDQ-SALSEX) [13,14], which has shown good psychometric properties. The first two questions use a yes/no format to record whether patients have noticed any change in their sexual function since they initiated treatment and whether the sexual dysfunction was spontaneously reported. The next four questions (items 1 to 4) employ a four-point scale, from no problem to severe problem, to assess the presence and severity of decreased libido, delayed ejaculation/orgasm, lack of ejaculation/orgasm and difficulties with having or maintaining erection/lubrication. The last question (item 5) evaluates the tolerability of the changes in sexual functioning on a four point scale: 0, No sexual dysfunction; 1, Well, no problem due to this reason; 2, Fair, the dysfunction bothers the patient, although they have not considered discontinuing the treatment for this reason, or it interferes with the couple’s relationship; 3, Poor, the dysfunction presents an important problem, and the patient has considered discontinuing treatment because of it, or it seriously interferes with the couple’s relationship. Items 1 to 5 account for the total score of the PRSexDQ-SALSEX, which ranges from 0 to 15. According to this total score, patients may be categorized as having no sexual dysfunction (a score of 0 or the item 1 (libido) scoring 1 and the item 5 (tolerability) scoring 1); mild dysfunction (total score of 3–5, provided that no item scores are greater than or equal to 2—i.e., provided that the patient does not have moderate sexual dysfunction in a specific dimension); moderate dysfunction (total score of 6–10 or an item scoring 2, provided that no item scores 3—i.e., provided that the patient does not have severe sexual dysfunction in a specific dimension); or severe sexual dysfunction (total score of 11–15 or an item scoring 3). 

The severity of sexual dysfunction was divided into 4 levels (no dysfunction, mild, moderate and severe) in both males and females.

### 2.4. Ethical Aspects

The study was previously approved by the Research Ethics Committee of Salamanca (CEIC). All patients signed their prior informed consent following the international norms and procedures of medical research in humans using the declaration of Helsinki of the World Medical Association of 1964.

### 2.5. Statistical Method

The main analysis was conducted with descriptive statistics from the sample. Afterwards, parametric and nonparametric tests were conducted according to the study’s distribution of variables. In addition, chi-square automatic interaction detection (CHAID) tests (chi-square AID, proposed by Kass in 1980) were used to determine predictive models. These trees follow an iterative and staged procedure, considering a qualitative dependent variable (in our case SD) from which categorical predictors will be obtained. For the statistical analysis, SPSS v.20 software (IBM, Armonk, NY, USA) was used.

## 3. Results

The final sample was composed of 256 subjects (188 males and 68 females) aged from 33 to 98 years (average = 59 years). The distribution by age was not homogenous. Most subjects were married with a stable partner with whom they were able to have sexual intercourse (80.4%). Many subjects (78.5%) perceived their global health to be good or very good, while 21.5% perceived it negatively (fair, bad or too bad). A statistical model was created to determine the probability of having dysfunction depending on the perceived general health before treatment. The probability of suffering from sexual dysfunction was 85.45% when the general health was perceived negatively before the onset of antihypertensive treatment.

Retroactive questions were also asked. The first was about how the participant considered their sex life to be before starting treatment. Most of them agreed that their previous sexual life was good (65.87%) or fair (19.71%). Finally, they were asked directly if they had noticed any change in their sexual function after starting the treatment. Almost half of the patients (43.7%) noticed some subjective deterioration in their sexual life (49.0% of males and 27.3% of females) after the treatment onset (*p*-value = 0.007).

### 3.1. Sexual Dysfunction Frequency in the Global Sample

Less than half of the patients (43.27%) had the subjective perception of having suffered changes in their sex life after beginning the treatment, while 56.73% experienced no change. Through the SALSEX questionnaire, 170/256 patients (66.40%) reported impaired sexual function and 86 patients (33.59%) showed no sexual dysfunction, with no significant statistical differences between males and females (*p*-value = 0.729). Of those that noted changes after commencing treatment, 96.7% displayed SD through the SALSEX, (*p*-value = 0.000); 27.3% of females and 49% of males reported negative changes in sexual function after treatment (*p*-value = 0.007).

### 3.2. Degree of Sexual Dysfunction in the Sample

To obtain greater clinical applicability, three degrees of SD classification were defined as follows: no dysfunction, mild dysfunction and clinically relevant dysfunction. This latest category was created unifying moderate and severe cases of SD detected by SALSEX. It was concluded that 123 subjects (48.05%) displayed clinically relevant dysfunction. There were no statistically significant differences between men and women (*p*-value=0.278). The analysis of the level of dysfunction is shown in Table 1.

Of the subjects that noted changes at the beginning of treatment, 7 subjects (7.8%) reported mild dysfunction and 80 (88.9%) reported clinically relevant dysfunction. Of the subjects that reported dysfunction, 72.2% reported good or very good sexual function before the antihypertensive treatment. There were no statistically significant differences between men and women.

### 3.3. Sexual Dysfunction in Different Phases of Sexual Response

Table 2 presents results regarding the way in which sexual dysfunction manifests according to the sexual response phase (items 1–4 of SALSEX).

### 3.4. Sexual Dysfunction and Treatments for Hypertension: Monotherapy vs. Combined Therapy

The sample was divided in two groups: monotherapy (40%) and combined treatment (60%). The treatments were prescribed with the following frequencies, according to drug family: diuretics (5.5%), beta blockers (6.30%), angiotensin-converting enzyme (ACE) inhibitors (3.5%), angiotensin receptor blockers (ARBs) (18.4%), calcium antagonists (5.9%) and others (0.4%). The most frequent combinations were diuretic + ACE inhibitor (10.2%), diuretic + ARB (12.1%), ARB + calcium antagonist (10.5%) and other treatments (11.3%), including combinations of more than three medicines. The results are described in Table 3.

All the treatments were associated with a high frequency of sexual dysfunction ranging from 53.3% to 83.9%. The combination of diuretic + ARB stands out, as 83.9% of patients reported sexual dysfunction; this was followed by ACE inhibitor monotherapy (77.8%). The compounds related with the least sexual dysfunction were the calcium antagonists (53.3%) and diuretics (57.1%).

In analyzing the contingency tables looking for any relation between monotherapy treatments and sexual dysfunction, there were no correlations in our sample. A significant relationship between combination diuretic + ARB therapy and sexual dysfunction was found (*p*-value = 0.028).

#### Clinically Relevant Sexual Dysfunction

To obtain a more practical clinical approach, mild sexual dysfunction was excluded from the analysis and moderate-to-severe sexual dysfunctions were combined into a group named clinically relevant sexual dysfunction (CRSD). The compounds showing higher CRSD were ACE inhibitors (77.8%), followed by the combination of diuretic + ARB (74.2% (*p*-value = 0.008)). Only 29.8% of patients on ARBs showed clinically relevant SD in monotherapy (Table 4).

**Figure 1:**
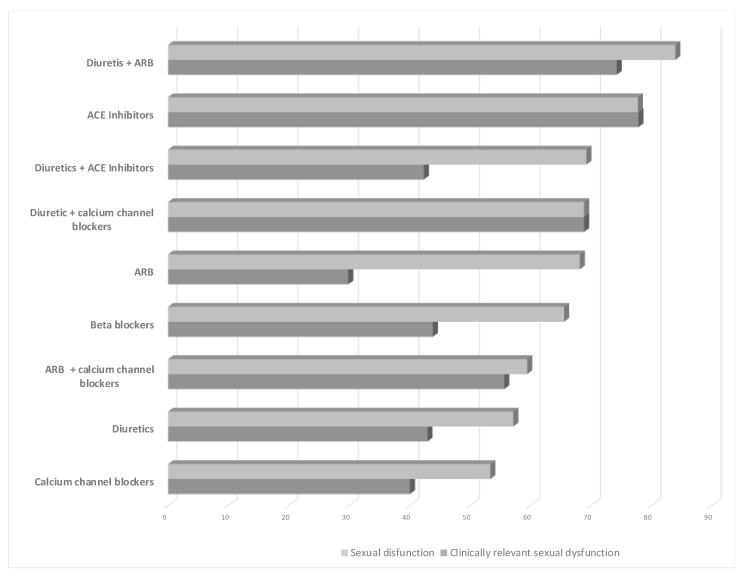
Percentage of patients who reported sexual dysfunction and clinically relevant sexual dysfunction (moderate or severe) for each type of treatment. ACE, angiotensin-converting enzyme; ARB, angiotensin receptor blocker.

Although there was also a significant relationship between the ARBs and sexual dysfunction (*p* = 0.000), in most cases, it produced mild dysfunction, as mentioned previously. The frequency of treatments’ association with SD and clinically relevant SD can be compared in Figure 1 for each type of drug.

### 3.5. Phases of Sexual Response

The desire phase: Overall, mild (27.8%) and moderate (35.6%) difficulties in desire were noted. Decreases in libido were observed in females (predominantly mild) and in males (majority moderate). Mild decreases were associated with ARB therapy, moderate dysfunction with ACE inhibitors and severe dysfunction was associated with beta blockers. A moderate decrease in libido was observed in every combined therapy except diuretic +ARB. These difficulties did not seem to be related to age, even though the 50–70-year-olds were the most affected group. The ACE inhibitors (55.51%) and the combinations ARB + calcium antagonist (50.1%), diuretics (35.7%) + calcium antagonists and diuretics (50.1%) and ARBs + diuretics (50.1%) were those that produced clinically relevant sexual dysfunction. ARBs alone were significantly related to sexual desire (*p*-value = 0.021), and the magnitude of decreased desire was the lowest among all monotherapies.

Ejaculation/orgasm delay: Most subjects reported having orgasm delay—mild (20%), moderate (31.1%) and severe (18.9%), predominantly in females. The association of diuretic + ARB showed the higher clinically relevant SD (35.5%), mainly in those aged under 50 years. The lowest clinically relevant SD was related with ARBs in monotherapy.

Inability to orgasm: Moderate anorgasmia was the most frequent symptom in females. In males, anorgasmia was experienced in a mild way, with more serious cases reported amongst those aged under 50 years. Mild cases seemed to be clearly influenced by the ARBs and the drug combination diuretic + ARB.

Clinically relevant sexual dysfunction was observed with the ACE inhibitors (55.5%) and the diuretic + calcium antagonist combination (43.8%). In the general sample, statistically significant differences were obtained for the diuretic + calcium antagonist combination (*p*-value = 0.031), but this group was too small to draw valid conclusions. Monotherapy with ACE inhibitors was significantly related to severe anorgasmia (*p*-value = 0.009) and the ARBs monotherapy group was the least related to anorgasmia (*p*-value = 0.007).

Erection/lubrication difficulties: The drugs that seemed to interfere most were the ACE inhibitors (66.6%), followed by the combined therapies diuretic + calcium antagonist (43.8%) and diuretic + ARB (43.8%). After analyzing lubrication difficulties amongst the women, significant relationships were found in the general sample for patients that were taking ARBs in monotherapy (*p*-value = 0.002). A high percentage of subjects with SD showed mild erection/lubrication difficulties (35.6%) and moderate difficulties (31.1%). Both sexes noted these difficulties, reporting similar difficulties in every age group. The ARBs appeared to cause mild dysfunction, as did all the combined therapies.

### 3.6. Drug Analysis According to the Changes in Relation to Previous Sexuality

Diuretics: On studying the 91 patients who took diuretics (alone or in combination), 17 showed poor previous sexual functioning. Of those, 3 (17.6%) improved following the onset of antihypertensive therapy, while 14 (82.4%) retained the dysfunction. Of those with good previous sexual activity, 55 (74.3%) displayed SD after treatment. Significant relationships were detected with the diuretics in monotherapy when previous sexuality was good or very good (*p*-value = 0.028).

Beta blockers: 52 patients received beta blockers. Of the 16 with poor sexuality before treatment, 12 (75%) worsened. Of the 36 with previous good sexuality, 24 (66.7%) presented SD. Of the 13 who had previous good sexuality, 8 (61.5%) developed SD. Beta blockers elicited SD both alone and in combination, regardless of prior sexuality.

ACE inhibitors: among 76 patients taking ACE inhibitors (alone or combined), 16 (21.1%) reported poor sexuality prior to treatment, and 60 good or very good. Of those 60, 37 (61.7%) showed SD. The p-value obtained by contrasting dysfunction with previous sexual health in only those taking ACE inhibitors (singularly or combined) gave no statistically significant result (*p*-value = 0.772). Of the nine subjects who were taking ACE inhibitors in monotherapy, only one had had previously poor sexuality. Of the eight remaining subjects, two continued to report good sexual activity after treatment, while six noted negative changes after ACE inhibitor treatment. Although the results are not conclusive due to the small sample size, it is evident that of those with previously good sexual activity, 75% experienced negative changes after treatment. 

ARBs: Of the 99 subjects under ARBs (singular or combined) and good/very good sexual health, 65 (60.7%) showed SD. Among the 25 with previous poor sexuality, 22 (88%) noted dysfunction (*p*-value = 0.024). Among the 47 who only took ARBs, 8 did not have previously good sexuality. Of these, the ratio no dysfunction/dysfunction was 25%:75%. In the 39 subjects who had good or very good previous sexuality, the ratio no dysfunction/dysfunction was 35.9%:64.1%. The differences were not statistically significant (*p*-value = 0.553). Unfortunately, the number of patients treated was very low, making it impossible to generalize the results.

Calcium antagonists: Despite there being a small number of patients in this group, significant ratios were obtained in those with poor previous sexual activity (*p*-value = 0.015). Of those with good previous sexuality, 61.5% reported SD. In the patients receiving combination therapy of calcium antagonists with other drugs, 14 subjects reported previously poor sexuality prior to commencing treatment. Of those 14, 4 appeared to improve, while 10 remained with dysfunction (71.4%). Of the 49 subjects in singular or combined calcium antagonist therapy, 49 reported previously good sexuality, and of those, 42.9% retained it, while it appeared to worsen in 28 subjects (57.1%). No statistically significant differences were seen.

Combination diuretic + ARB: Among the 31 subjects under this combination, no association with previous sexuality was detected. Six subjects did not have satisfactory sexual activity, and only one reported an absence of dysfunction after treatment, while in five (83.3%), the dysfunction remained. Of the 25 with satisfactory sexuality, 22 (88%) displayed dysfunction. Therefore, the combination of diuretic + ARB was shown to worsen perceived sexuality, as much amongst those that were previously satisfied as among those that were not. It appears that these variables are dependent on each other and were statistically related when previous sexuality was good or very good (*p*-value = 0.009). In the created statistical model, the odds ratio for the variable diuretic + ARB was 2.925. Therefore, the ratio among hypertensive subjects suffering from sexual dysfunction versus those who do not was 2.92, 5 times higher in patients who took diuretic + ARB therapy compared to those who did not. This association was statistically significant (*p* = 0.035).

### 3.7. Accepting Sexual Dysfunction

Item 5 of the SALSEX questionnaire measures patients’ acceptance of sexual dysfunction after its appearance. The majority of the subjects that noted changes after the treatment accepted it poorly or near poorly, with a higher proportion in males (74.3% males/53.3% females (*p*-value = 0.049). Age appeared to have no correlation with the acceptance of SD (*p*-value = 0.627).

### 3.8. Relationship of SD with Other Comorbid Illnesses

A significant statistical association appeared between patients suffering from clinically relevant SD and those with coronary and musculoskeletal pathology (Table 5). We created two statistical models and found that SD was 14 times more likely to present as a comorbidity with coronary disease than not to present it. On the other hand, the probability of developing SD in patients suffering from musculoskeletal disease was 88.23%. For the rest of the concomitant illnesses, it appeared that subjects did not present many cases, irrespective of their scope. Obesity was the only variable that appeared to be related to sex (*p*-value = 0.026).

### 3.9. Sexual Dysfunction and Age

For our statistical analysis, the subjects were split into four age groups (under 51, 51–50, 61–70 and above 70). The highest percentage of cases with SD occurred in those aged 51–70 years. SD was related to age in the following sexual response phases: decreased desire (*p*-value = 0.015); orgasm delay (*p*-value = 0.027); and inability to have orgasms or ejaculate (*p*-value = 0.024). Subjects under 70 years of age were the most dissatisfied with the worsening of sexual functioning, especially when the SD was moderate, which usually appeared between the ages of 51 and 60.

#### Predictive Models with Segmentation Trees

According to the model, the best predictor when looking for the subjects’ profiles with different patterns was the patient’s general health (*p*-value = 0.001). Those whose general health was poor were more likely to suffer from SD. In those over 60 years of age who were in good health, the greatest predictor of SD was coronary disease (*p*-value = 0.021). In all subjects with coronary pathology there was clinically relevant SD. In another segment the best predictor was musculoskeletal pathology (*p*-value = 0.014). All of those suffering from musculoskeletal pathology showed clinically relevant SD. In those without this pathology, the best predictor was the combined treatment diuretic + ARB (*p*-value = 0.011). Of those taking this combination, 76.5% had clinically relevant SD, while, in the other group, 43.7% had no dysfunction. According to this model, we identified the following three groups of higher risk:

Group 1 (*n* = 14): Healthy subjects, over 60 years of age, with either coronary disease or musculoskeletal pathology—100% suffered from clinically relevant SD;

Group 2 (*n* = 17): Healthy subjects, over 60 years of age, without coronary pathology or musculoskeletal problems, taking a combination of diuretic + ARB—76.5% suffered from clinically relevant SD;

Group 3 (*n* = 27): Subjects with poor general health, of which the degree of hypertension was not known—88.9% had clinically relevant SD.

## 4. Discussion

HBP is a common pathology associated with heterogeneous biological and lifestyle risk factors that can significantly influence sexual function and the quality of life of patients and their partners [5,15,16]. Research over the past 30 years examining sexual functioning in patients with increased blood pressure has described a great variability in the frequency of SD associated with some antihypertensive treatments [4,5,8,10,11,17,18,19]. The different mechanisms of action inherent in each group of drugs and the difficulties patients experience in spontaneously reporting sexual adverse effects undoubtedly contribute to increase the difficulty in properly estimating the magnitude of this problem.

In this study, the frequency of SD in patients on antihypertensive treatment was quantified through the SALSEX questionnaire (widely used to measure iatrogenic sexual dysfunction due to pharmacological treatments), analyzing different variables that may interfere with sexual response. Due to the low spontaneous reporting of SD in our sample (6.81% of females and 24.8% of males), it would be necessary to systematically carry out a specific anamnesis including data on sexual functioning as accurately as possible through validated questionnaires with the aim of clarifying patients’ current sexual status before and after the treatment onset.

This adverse effect has previously been more frequently studied in males, and little research shows differences in prevalence between men and women [17], despite the fact that females seem to suffer from sexual dysfunction at a higher or equal rate than men treated with antihypertensives [6,11,20]. Our sample had a greater proportion of males, and although there were no significant differences in the frequency of SD between genders, symptoms were more severe in males. Our data are in partial agreement with previous studies reporting no difference in SD in females on antihypertensive treatment compared to control groups [18]. Contradictory findings in this subject have been observed due to differences in methodology and inclusion/exclusion criteria in the scientific literature.

The fact that our sample is mostly represented by men may be explained by the influence of greater physical and cultural risk factors of SD associated with the male gender (e.g., tobacco and alcohol abuse, obesity, sedentary lifestyle). A notable difference between the spontaneous communication of SD and the higher frequencies obtained through the SALSEX questionnaire was found. These differences could be explained by the fact that, in general, the medical staff did not routinely interview their patients about previous sexual functioning and satisfaction; this was especially true for patients who were elderly, widowed, accompanied by a relative or recently admitted. Of the respondents, 80.4% were male, married and with a stable partner. These data show that certain cultural taboos may remain regarding female sexuality, and it would be desirable to increase the training for clinicians and health providers in dealing with sexual dysfunction in females.

Clinical studies about patient perceived general health as a predictor of SD are very scarce. In our sample, 78.5% of the subjects perceived their general health to be good or very good, and this correlated with a positive effect on their sexuality. This fact could be interpreted not only in the context of the biological implications but also as it relates to the patient’s better attitude towards their sexuality.

Although there are numerous previous studies on the prevalence of SD with antihypertensive treatment, the different stages of sexual response have hardly been evaluated [21], with most studies focusing on erectile dysfunction. In our study, most subjects reported mild erection/lubrication difficulties, and a high percentage reported delayed orgasm and/or anorgasmia/anejaculation. Subjects who noticed any symptoms of iatrogenic SD clearly related them to the new antihypertensive treatment.

From a practical point of view, in order to help clinicians in making decisions on the management of adverse effects, specific recommendations should be addressed in patients with clinically relevant SD—those with moderate or severe SD and with SD that is poorly tolerated. Most of the subjects with SD on monotherapy with ARBs reported a frequent but mild sexual impairment, so this group seems to be the best option to avoid relevant sexual dysfunction from the beginning [22,23]. This fact should influence the recommendations of the clinical guidelines [24], with the aim of improving sexual dysfunction [12]. On the other hand, the combination of diuretic + ARB strongly worsened sexual functioning, with a predominance of clinically relevant SD. Therefore, clinicians should consider avoiding this prescription as a first option in patients with good sexual functioning before the diagnosis of increased blood pressure. Quality of life deterioration and poor tolerance of the treatment have also been described by other authors [25]. Additionally, ACE inhibitors in monotherapy showed a moderate-to-severe decrease in sexual functioning, including low desire, delayed orgasm, anorgasmia/anejaculation and arousal dysfunction in both males and females. This should be considered alongside the fact that they can produce other negative effects in reproduction [21], which contradicts some previous studies showing a neutral or beneficial effect on sexual activity [21,22,23,26,27,28].

Age seemed to be a key factor in the presence of SD and in the acceptance of the SD. Low desire and anorgasmia/anejaculation were significantly more intense in patients aged 51–70 years, as part of the natural process of aging [29,30,31,32] The duration of the iatrogenic SD may therefore be crucial, as it is known that, in many cases, the occurrence of side effects may also be related to the duration of treatments for chronic diseases [33,34,35]. However, studies which report results describing the effects of either discontinuing treatment or switching to other drugs on SD are lacking.

A novel aspect of our study is that the frequency of the spontaneous reporting of SD (item B of the SALSEX questionnaire) was only 14.8% (four times higher in men than in women). A significantly higher frequency of this adverse event (66.4%) was obtained through the SALSEX questionnaire, indicating a large under-reporting of SD when patients were not directly questioned on sexual functioning.

The risk of treatment abandonment in patients with severe SD and poor tolerance is highly relevant, especially in men, with some previous studies analyzing this risk [18]. The most poorly tolerated treatments (i.e., patients with severe SD who expressed a desire to stop treatment due to SD) were the monotherapies with calcium antagonists (26.7%), and combination therapies of diuretic + calcium antagonist (25%, *p* = 0.028) and diuretic + ARB (22.6%, *p* = 0.020). In contrast, subjects on monotherapy with ARBs (*p* = 0.013) or ACE inhibitors, whose severity of SD was found to be mild, showed no risk for a lack of adherence. Because the abandonment of treatment is accompanied by very negative consequences on the overall health of patients, these results highlight the clinical need for specific and targeted sexual functioning interviews before and after initiating any antihypertensive treatment. Due to the large differences observed in the frequency of SD with the different compounds, and considering the similar efficacy of all of them, it would be reasonable to choose the antihypertensive considering the patient’s quality of life and respect for their previous sexual life. Furthermore, in the light of current taboos among health workers and patients in the assessment of sexuality in daily clinical practice, it would be reasonable to improve the doctor/nurse–patient relationship by promoting more appropriate communication strategies that allow the early detection of any sexual disorder associated with the prescription of an antihypertensive compound. This strategy would certainly improve the adherence to treatment and the quality of life of patients and their partners.

Finally, the frequency of SD with all treatments tested was high, ranging from 53.3% to 83.9%. However, the intensity of SD should also be analyzed in order to improve the perspective of the sexual function impairment. Monotherapy with a calcium antagonist was the least damaging treatment, with an SD frequency of 53.3%, similar to what has been reported in other studies [23,31,36].

## 5. Limitations

Although the global sample size was initially sufficient (256 patients), some of the treatment subgroups were very small; therefore, the generalizability and validity of the results may be compromised. The presence of a larger sample for each treatment group would undoubtedly improve the results of the statistical analysis. Additionally, there was a lack of a control group, which could limit the conclusions of the study. There is great difficulty in finding an adequate control group due to the heterogeneous characteristics of the sample in terms of its demographic, clinical, comorbidity and concomitant treatment characteristics. However, the differences found with the different drugs after the statistical analysis carried out could serve as a guide to the clinician to make decisions based on real-life observation.

However, the presence of inclusion and exclusion criteria, excluding those patients with sexual dysfunction prior to the treatment onset, was intended as a control to eliminate false positives. Nevertheless, this naturalistic, observational and cross-sectional design allows us to obtain a very rough estimate in real clinical practice following the same design with which relevant results have been obtained in large patient samples using the SALSEX questionnaire to measure SD frequency with other drugs and different pathologies (e.g., antidepressants and antipsychotics) [36,37,38,39].

Future comparative research in larger samples would be desirable to clarify SD frequencies using a more appropriate design to confirm these results.

On the other hand, patient data were not collected consecutively, as it was left to the initiative of the participating researchers to conduct the interview. Therefore, it is possible that an undetermined number of subjects were not investigated because of social taboos or for other reasons. Routine clinical practice studies may be biased in terms of the generality and specificity of the data; however, they show results that more closely resemble the real world of everyday clinical practice. It would be desirable to improve the training of health workers and to standardize the interviewing of sexual aspects of patients, especially in chronic patients and patients with other associated diseases, as the deterioration of their sexual lives is likely to be even greater.

## 6. Conclusions

Sexual dysfunction caused by antihypertensive drugs, in any of its manifestations (low desire, delayed orgasm, anorgasmia/anejaculation and arousal difficulties) was found to be very frequent (66.4% on average, range from 53.3 to 83.9%) in this study, when measured with a specific questionnaire, the PRSexDQ-SALSEX. Nevertheless, in clinical practice, SD could be underestimated because spontaneous reporting was low (14.8%), although men reported sexual dysfunction four times more often than women. Combination therapy was associated with higher percentages of SD than monotherapy. The most sexually damaging treatment groups were diuretic + ARB (83.9%) and ACE inhibitors (77.8%), affecting all sexual phases: decreased desire, delayed orgasm/ejaculation, anorgasmia/anejaculation and erection/lubrication difficulties. However, monotherapeutic ARBs treatment was associated with a high percentage of SD (68.1%), although most cases were mild and not clinically relevant. Calcium antagonist monotherapy had the lowest percentage of SD (53.3%). SD depends on the subjective assessment of previous sexuality, as 88.9% of those who noticed changes presented clinically relevant SD—most of them being men. The most poorly tolerated treatments were the combinations of diuretic + calcium antagonist and diuretic + ARB, since 25% of patients on these regimens had considered abandoning treatment. However, monotherapy with ARBs and ACE inhibitors did not present adherence problems.

Gender appeared to be independent of SD, although females showed milder SD than males. SD was more prevalent in older age. Finally, according to predictive models using CHAID trees, the highest risk of developing SD was found in patients with poor general health, aged over 60 years, with concomitant coronary or musculoskeletal pathology or comorbid mood disorders and those taking the therapeutic combination diuretic + ARB. 

## Figures and Tables

**Table 1:** Degree of Sexual Dysfunction in the sample: SALSEX questionnaire scores.

Degree of SD	Total *n* = 256 *n* (%)	Female *n* = 68 *n* (%)	Male *n* = 188 *n* (%)
Mild dysfunction	47 (18.36)	17 (25)	30 (16.95)
Moderate dysfunction	91 (35.55)	19 (27.94)	72 (38.29)
Severe dysfunction	32 (12.5)	8 (11.76)	24 (12.76)
Clinically relevant dysfunction *	123 (48,05)	27 (39.70)	96 (51.05)

* This category included severe and moderate cases of SD detected by SALSEX Questionnaire.

**Table 2:** Frequency of sexual dysfunction in the global sample. SALSEX questionnaire (items 1, 2, 3 and 4).

	Females %	Males %	*p*-Value
Decrease in desire	55.8	54.2	0.385
Orgasm/ejaculation delay	42.6	45.7	0.533
Inability to ejaculate/orgasm	42.6	43.6	0.533
Erectile/lubrication difficulties	52.9	59.6	0.715

**Table 3:** Antihypertensives and frequency of sexual dysfunction.

	*n*	SD %	Decrease in Desire %	Orgasm/Ejaculation Delay %	Inability to Ejaculate/Orgasm %	Ejaculation/Lubrication Difficulties %
Monotherapy						
ACE inhibitors	10	77.8	55.51	66.6	55.5	77.7
ARBs	47	68.1	48.9	44.7	46.8	57.4
Beta blockers	16	65.5	43.9	43.8	43.8	62.6
Diuretics	15	57.1	57.1	35.6	35.7	49.9
Calcium antagonists	15	53.3	33.4	43.1	20	46.6
Combined therapy						
* Diuretic + ARB	31	83.9	71	58.1	51.6	70.9
Diuretic + ACE inhibitor	26	69.2	42.2	46.01	42.3	61.6
Diuretic + calcium antagonist	16	68.8	62.6	62.6	62.6	68.8
ARBs + calcium antagonist	27	59.4	51.8	37	33.3	51.8

* *p* < 0.028 (CI 95%). ACE, angiotensin-converting enzyme; ARBs, angiotensin receptor blockers.

**Table 4:** Clinically relevant SD and treatments for hypertension.

	*n*	Clinically Relevant SD %	Decrease in Desire %	Orgasm/Ejaculation Delay %	Inability to Ejaculate/Orgasm %	Ejaculation/Lubrication Difficulties %
Monotherapy						
ACE inhibitors	10	77.8	55.51	44.4	55.5	66.6
Beta blockers	16	43.8	25.1	31.3	25	31.3
Diuretics	15	42.9	35.7	28.5	14.3	14.2
Calcium antagonists	15	40	13.4	36.4	6.7	33.3
ARBs	47	29.8	14.9	14.9	4.2	10.6
Combined therapy						
*Diuretic + ARB	31	74.2	45.2	35.5	22.6	41.9
Diuretic + calcium antagonist	16	68.8	50.1	50.1	43.8	43.8
ARB + calcium antagonist	27	55.6	33.3	25.9	22.2	25.9
Diuretic + ACE inhibitor	26	42.3	23	26.9	19.2	30.8

* *p* < 0.05 (CI 95%).

**Table 5:** Relation between sexual dysfunction and other pathologies.

Comorbid Disease	*p*-Value	*n*
Coronary	0.023 *	15
Musculoskeletal	0.049 *	17
Diabetes	0.299	51
Cardiological (not coronary)^†^	0.650	11
Endocrine	0.682	14
Oncological	0.374	6
Kidney failure	0.152	4
Hepatic failure	0.313	2
Peripheral vascular	0.599	7
State of mind/Mood	0.180	20
Other pathologies	0.007 *	19

* *p* < 0.05 (CI 95%); ^†^ valvular disease, heart failure or arrhythmia.
